# Effect of YAP/TAZ on megakaryocyte differentiation and platelet production

**DOI:** 10.1042/BSR20201780

**Published:** 2020-08-20

**Authors:** Chanchao Lorthongpanich, Nittaya Jiamvoraphong, Phatchanat Klaihmon, Usaneeporn Lueangamornnara, Yaowalak U-pratya, Chuti Laowtammathron, Surapol Issaragrisil

**Affiliations:** 1 Siriraj Center of Excellence for Stem Cell Research, Department of Medicine, Faculty of Medicine Siriraj Hospital, Mahidol University, Bangkok, Thailand; 2 Division of Hematology, Department of Medicine, Faculty of Medicine Siriraj Hospital, Mahidol University, Bangkok, Thailand; 3 Bangkok Hematology Center, Wattanosoth Hospital, BDMS Center of Excellence for Cancer, Bangkok, Thailand

**Keywords:** Hippo pathway, Megakaryocyte, Platelet, YAP

## Abstract

Platelet transfusion is required for life-threatening thrombocytopenic bleeding, and single donor platelet concentrate is the ideal transfusion product. However, due to the inadequate number of donors that can donate a large volume of platelets, *in vitro* platelets production could be an alternative. We developed an *in vitro* production system designed to increase the platelet production yield from cultured cells. Previously, we reported that depletion of a Hippo pathway core kinase (LATS1/2) inhibited platelet production from cultured megakaryocytes. In the present study, we further investigated the role of the Hippo pathway in megakaryocyte proliferation and platelet production by focusing on the role of its effector proteins (YAP and TAZ), which are down-stream targets of LATS1/2 kinase. We found that YAP plays an essential role in megakaryoblastic cell proliferation, maturation, and platelet production, while TAZ showed minor effect. Knockdown of YAP, either by genetic manipulation or pharmaceutical molecule, significantly increased caspase-3-mediated apoptosis in cultured megakaryocytes, and increased platelet production as opposed to overexpressing YAP. We, therefore, demonstrate a paradigm for the regulation of megakaryocyte development and platelet production via the Hippo signaling pathway, and suggest the potential use of an FDA-approved drug to induce higher platelet production in cultured cells.

## Introduction

Patients with life-threatening thrombocytopenic bleeding require platelet transfusion support. Single donor rather than pooled platelet is the most ideal transfusion product choice because the likelihood of developing alloimmunization and/or infection is lower. An approach of current interest is *in vitro* production of platelets from a single donor.

It was estimated that one megakaryocyte could generate and release a few thousand platelets *in vivo* [1–3]. However, poor platelet release was commonly observed when megakaryocytes were cultured *in vitro*. Better understanding of the factor(s) that affect megakaryocyte proliferation and differentiation is needed to facilitate the development of a new culture system to enhance platelet-like particle (PLP) production from cultured megakaryocytes.

We previously demonstrated that Hippo pathway core kinase LATS1/2 plays an essential role in regulating megakaryocyte proliferation and differentiation [4]. In the present study, we further explored the role of the Hippo effector proteins YAP and TAZ on megakaryocyte differentiation and platelet production.

## Materials and methods

### Cell and cell culture

Megakaryoblastic leukemia cell line (MEG-01) was purchased from ATCC (Manassas, VA, U.S.A.), cultured in Roswell Park Memorial Institute (RPMI) 1640 medium (Gibco; Thermo Fisher Scientific, Waltham, MA, U.S.A.) supplemented with 10% fetal bovine serum (FBS; Merck Millipore, Darmstadt, Germany) at 37°C in a humidified atmosphere of 5% CO_2_ in air. Cells were cultured at a seeding of density 1 × 10^5^ cells/ml, and then subcultured every 2 days.

### Generation of YAP and TAZ knockdown (KD) cells

The pSuper-Retro-puro YAP plasmid [5] was kindly donated by Dr Chan Siew Wee of the Institute of Molecular and Cell Biology, Singapore. For the preparation of retrovirus, the pSuper-Retro-puro YAP plasmid was transfected using Lipofectamine 3000 (Life Technologies, Carlsbad, CA, U.S.A.) into 4 × 10^6^ Plat-A cells in a 100 mm cell culture dish. After 24 h, the medium was changed to fresh Plat-A cell medium comprised of Dulbecco’s Modified Eagle Medium (DMEM; Gibco) + 10% FBS. Virus-containing supernatants were collected at day 2 after transfection, passed through a 0.45-µM filter (Jet Biofil, Guangzhou, China), and concentrated by transferring the virus-containing supernatant through Amicon Ultra-15 Centrifugal Filter Units (Merck Millipore, Berlin, Germany) with subsequent centrifugation at 4,000 ***g*** for 30 min at 4°C. The concentrated virus was collected and added to 2.5 × 10^5^ MEG-01 cells plated in a 12-well plate in RPMI with 10% FBS in the presence of 5 µg/ml Polybrene (Sigma-Aldrich, St. Louis, MO, U.S.A.). The medium was changed the next day to RPMI with 10% FBS. This cell line was thereafter referred to as shYAP. TAZ knockdown plasmid was purchased from GenScript.

### Generation of YAP-overexpressing cells

The site-directed mutagenesis YAP^S127A^ and YAP^S5A^ constructs [6] were transfected using 4D-Nucleofector™ (Lonza, Basel, Switzerland). Overexpression was confirmed by Western blotting. These cell lines were thereafter referred to as S127A and S5A for YAP^S127A^ and YAP^S5A^, respectively.

### Quantitative-PCR and data analysis

Isolated total RNA was reverse transcribed with the High-Capacity cDNA Reverse Transcription Kit (Applied Biosystems, Foster City, CA, U.S.A.). Quantitative real-time polymerase chain reaction (PCR) was performed using Realtime PCR Master Mix (Applied Biosystems) and the Universal Probe Library (UPL; Roche Life Science, Penzberg, Germany) in a final volume of 10 μl. Real-time PCR assays were performed using a CFX384 Touch Real-Time PCR Detection System (Bio-Rad Laboratories, Hercules, CA, U.S.A.).

### Western blot analysis

The presence of LATS1, LATS2, YAP, pro-caspase-3, cleaved-caspase-3, and the c-Myc proteins was determined by Western blotting. Total protein was extracted from cells using a protein lysis buffer (10× RIPA: Cell Signaling Technology, Danvers, MA, U.S.A.) containing protease inhibitors (Roche Life Science). The denatured protein was run onto (7–12%) SDS/polyacrylamide gels, and the separated proteins were transferred to PVDF membranes (Merck Millipore, Berlin, Germany) and probed with the following primary antibodies: rabbit anti-LATS1 (Cell Signaling Technology), anti-LATS2 (Cell Signaling Technology), anti-YAP (Cell Signaling Technology), anti-phosphorylated (p)-YAP (Cell Signaling Technology), anti-caspase-3 (Cell Signaling Technology), all diluted 1:1000; and, anti-β-actin-peroxidase (ACTB; Sigma-Aldrich, St. Louis, MO, U.S.A.) diluted 1:25,000. Peroxidase-conjugated, species-appropriate antibody at 1:5000 dilution was added, with subsequent detection by autoradiography using enhanced chemoluminescence (Merck Millipore). ACTB served as the loading control.

### Platelet-like particle (PLP) collection and count

MEG-01cells were cultured at a density of 10^5^ cells/ml for 3–5 days before harvesting. The supernatant was collected after centrifugation at 800 revolutions per minute (rpm) for 10 min. For platelet precipitation, the collected supernatant was concentrated by centrifugation at 1,500 rpm for 5 min, the platelets were resuspended with PBS to 200 µl, and counting was performed using an automatic complete blood count (CBC) analyzer (XS-800i; Sysmex, Kobe, Japan).

### Flow cytometric analysis

To determine the expression of megakaryocyte and platelet-related markers, 100 µl of the cell suspensions were incubated with 5 µl of PE/Cy7-conjugated anti-human CD41/61 antibody (BioLegend, San Diego, CA, U.S.A.) and 5 µl of APC-conjugated anti-human CD41 antibody (BioLegend) for 30 min at 4°C, and then resuspended in 300 µl of 1% paraformaldehyde in phosphate-buffered saline (PBS). Megakaryocyte and platelet-like particles (PLP) were defined using the same log forward (FCS-A) and side scatter (SSC-A) properties as those obtained by measurement of platelets from human peripheral blood. Megakaryocyte maturity was evaluated by changes in CD41 cell-surface expression, as well as by changes in their forward and side scatter properties.

### Platelet microaggregation

In the present study, we set forth to determine the efficiency of PLPs derived from small molecule-treated cells; therefore, only self-aggregation was evaluated, and no human blood platelets were added to enhance the aggregation processes. Platelet microaggregation assay was performed according to Lu et al. [7] with slight modifications. Briefly, cells were centrifuged at 800 rpm for 10 min, after which the supernatant was transferred into a 5 ml polystyrene round bottom tube (BD Falcon; BD Biosciences, Franklin Lakes, NJ, U.S.A.) and centrifuged at 2,500 rpm for 10 min. The platelets in the precipitated fraction were counted and adjusted to 3 × 10^5^ platelets in a total volume of 250 µl. Thrombin (0.5 U/ml) was added and mixed at 1,200 rpm at a temperature of 37°C to activate platelet aggregation. Platelet microaggregates were spread onto glass slides and visualized by confocal fluorescence microscopy (Nikon NIS-Elements; Nikon Instruments, Tokyo, Japan).

### Small molecule preparation and treatment

Valproic acid (VPA), verteporfin (VP), dobutamine (DH), and lysophosphatidic acid (LPA) were purchased from Sigma-Aldrich and prepared as 20 mmol/l stock solutions in the appropriate diluent. The stock solutions were then aliquoted and maintained at −20°C until use. Cells treated with verteporfin were protected from light at all times to prevent photosensitization.

### Statistical analysis

Independent paired Student’s *t*-test was used to compare platelet differentiation between control and different groups. For CD41^+^ platelet count analysis, thrombocytic parameter-matched events were compared. Mann–Whitney *U* test was used to compare non-parametric variations between groups. A *P*-value of <0.05 was considered to be statistically significant. All results are presented as mean ± standard error of mean (SEM)***.***

## Results

### Knockdown of YAP/TAZ on CD41^+^ cells, and PLP production

**Figure 1:**
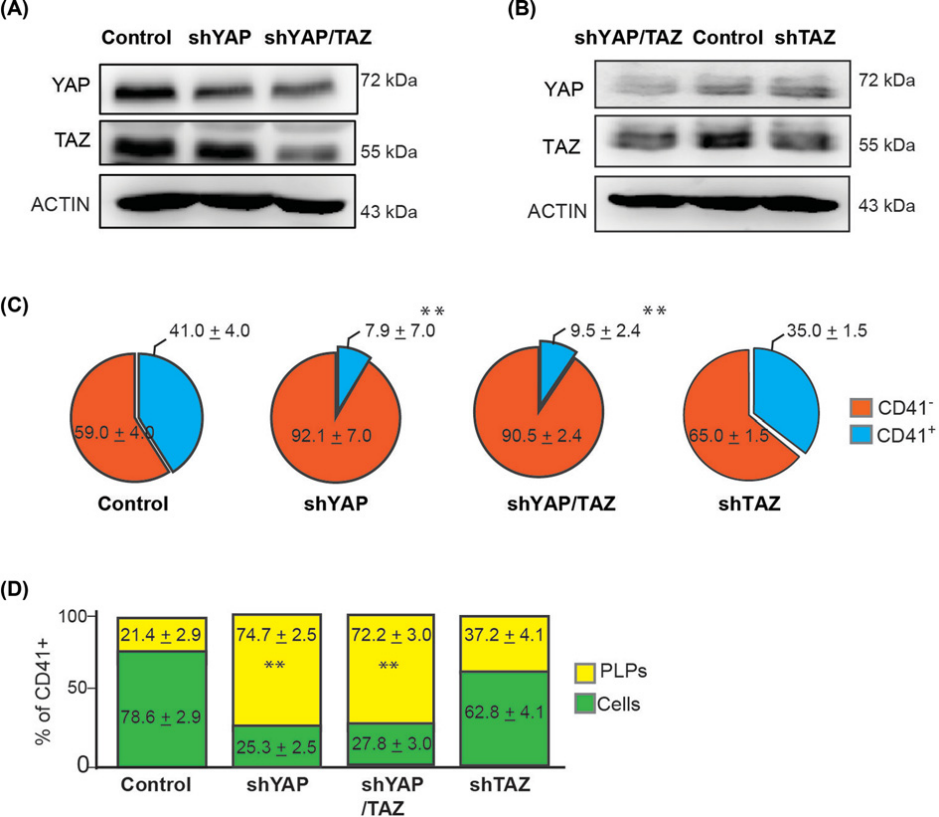
Effect of *YAP* and *TAZ* on the volume of CD41+ cell and platelet-like particle (PLP) production (**A**) Western blot analysis confirmed reduction of YAP and TAZ at 24h after targeting by shYAP, shYAP/TAZ, and (**B**) shTAZ plasmids. (**C**) Pie chart showing the proportion of CD41^−^ and CD41^+^ cells in naïve MEG-01 control cells (MEG-01-shYAP, MEG-01-shYAP/TAZ, and MEG-01-shTAZ). (**D**) Bar chart showing the proportion of megakaryocytes and PLPs derived from the CD41^+^ population in each sample. Cells transfected with an empty vector plasmid served as a control (**P<0.01; Mann–Whitney *U* test). Full Western blot images associated with Figure 1 can be found in Supplementary Figures S1–3.

YAP and TAZ are two important effectors of Hippo pathway signaling. These two proteins have been reported to play redundant roles in many cell types, including HEK293 [8] and mouse embryo [9]. We generated YAP-KD, TAZ-KD, and YAP/TAZ KD cell lines in MEG-01 cells, and used them to determine the effect of YAP and/or TAZ on megakaryoblastic cell differentiation and platelet production. A significant reduction of YAP was observed in YAP-knockdown cells, and reduction of both YAP and TAZ was observed when a plasmid targeting both *YAP* and *TAZ* (shYAP/TAZ) was introduced into these cells (Figure 1A,B). CD41, which is a common marker for megakaryocyte and platelets, is expressed on the plasma membrane of mature megakaryocyte cells prior to platelet release [10,11]. Our previous work and other studies demonstrated that 20–40% of a population of MEG-01 cells is made up of CD41^+^ cells, and MEG-01 cells can produce platelet-like particles (PLPs) either spontaneously or upon treatment with platelet induction agents, such as valproic acid (VPA) [4,12]. In this experiment, the proportions of CD41^−^ and CD41^+^ cells in each sample were counted and shown in the pie chart to indicate the differentiation capacity of MEG-01 after YAP and/or TAZ were depleted (Figure 1C; pie charts show an average value from three independent experiments with 30,000 events per experiment). Knockdown of YAP and double knockdown of YAP and TAZ (shYAP/TAZ) significantly reduced the number of CD41^+^ cells. Interestingly, knockdown of TAZ alone does not effectuate a dramatic reduction of CD41^+^ cells when compared with the effect of treatment by shYAP or shYAP/TAZ (Figure 1C), which suggests that expression of YAP influences the amount of CD41^+^ cells.

To determine whether the CD41^+^ population are megakaryocyte cells and PLPs, we used the same parameters as gating regions (log forward and SSC-A) as those used for platelets derived from human peripheral blood. We found an increasing amount of CD41^+^ PLPs in YAP and YAP/TAZ knockdown cells (Figure 1D), whereas TAZ knockdown cells had only minor effect on both megakaryocyte differentiation and platelet production. We, therefore, explored the effect of YAP rather than TAZ in subsequent experiments.

### Forced-expression of YAP in MEG-01 increases CD41^+^ cells, but reduces CD41^+^ PLP release

**Figure 2:**
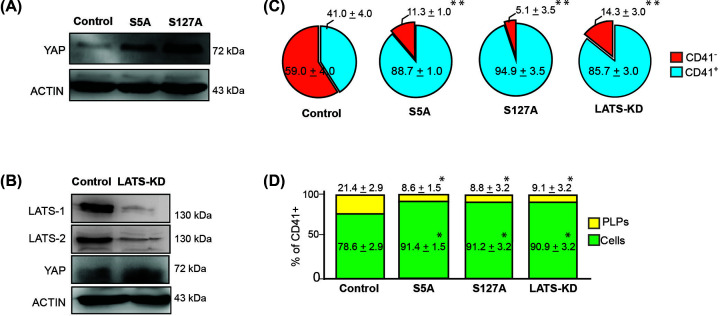
Overexpression of *YAP* increases CD41+ cells, but inhibits platelet-like particle (PLP) release (**A**) Western blot analysis confirmed overexpression of YAP at 24 h after YAP^S5A^ or YAP^S127A^ or empty vector plasmid backbone (control) introduction into MEG-01 cells. (**B**) Reduction of LATS1 and LATS2, and increase of YAP in LATS-KD MEG-01 cell line. (**C**) Pie chart showing the proportion of CD41^−^ and CD41^+^ cells in naïve MEG-01 control cells, and in MEG-01-YAP^S5A^, -YAP^S127A^, and -LATS-KD cell lines (Representative data from 3 independent experiments with 50,000 recorded events per experiment). (**D**) Bar chart showing the proportion of megakaryocytes and PLPs derived from the CD41^+^ population in YAP overexpressing cells. Cells transfected with an empty vector plasmid served as a control (*P<0.05, **P<0.01; Mann–Whitney *U* test). Full Western blot images associated with Figure 2 can be found in Supplementary Figures S4 and 5.

To confirm that YAP is required for megakaryoblastic cell differentiation, we performed gain-of-function experiments in MEG-01 cells via transfection with wild-type YAP, or site-directed mutagenized YAP^S5A^ or YAP^S127A^ to constitutively generate active form of YAP, or indirectly increasing endogenous YAP by inactivating the LATS1/2 kinases (LATS-KD) [4]. Significant up-regulation of YAP was observed after MEG-01 cells were transfected with YAP plasmids. However, with unclear mechanism MEG-01 cells transfected with wild-type YAP plasmids were gradually died in culture. We therefore excluded the wild-type YAP from the experiment. Interestingly, MEG-01 cells transfected with site-directed mutagenized YAP^S5A^ or YAP^S127A^ were able to grow and expand in culture. Significant up-regulation of YAP was observed in YAP^S5A^ or YAP^S127A^ transfected cells (Figure 2A). Down-regulation of both LATS1 and LATS2, but up-regulation of YAP was observed in the LATS-KD cell line (Figure 2B). Floating, adherent, and anucleated PLPs from YAP^S5A^, YAP^S127A^, LATS-KD, and naïve MEG-01 cells were collected and tested for CD41 expression using flow cytometry. An unstained sample served as the negative control. The proportion of CD41^−^ and CD41^+^ cells in each sample is shown in the pie chart in Figure 2C. Overexpression of YAP, (YAP^S5A^, YAP^S127A^, and LATS-KD) significantly increased the number of CD41^+^ cells when compared with control cells (88.7 ± 1.0%, 94.9 ± 3.5%, 85.7 ± 3.0%, and 41.0 ± 4.0%, respectively; *P*<0.01).

Although a high percentage of CD41^+^ cells was observed in YAP^S5A^, YAP^S127A^, and LATS-KD, these cells were found to produce significantly less CD41^+^-PLPs when compared with control MEG-01 cells (8.6 ± 1.5%, 8.8 ± 3.2%, 9.1 ± 3.2%, and 21.4 ± 2.9%, respectively; *P*<0.01; Figure 2D). Our results indicate that overexpression of YAP facilitates differentiation of MEG-01 cells into CD41^+^ cells, but diminishes platelet production, which confirms that YAP regulates megakaryocyte differentiation and platelet production.

### Expression of YAP on anti-apoptotic gene expression and megakaryocyte differentiation

**Figure 3:**
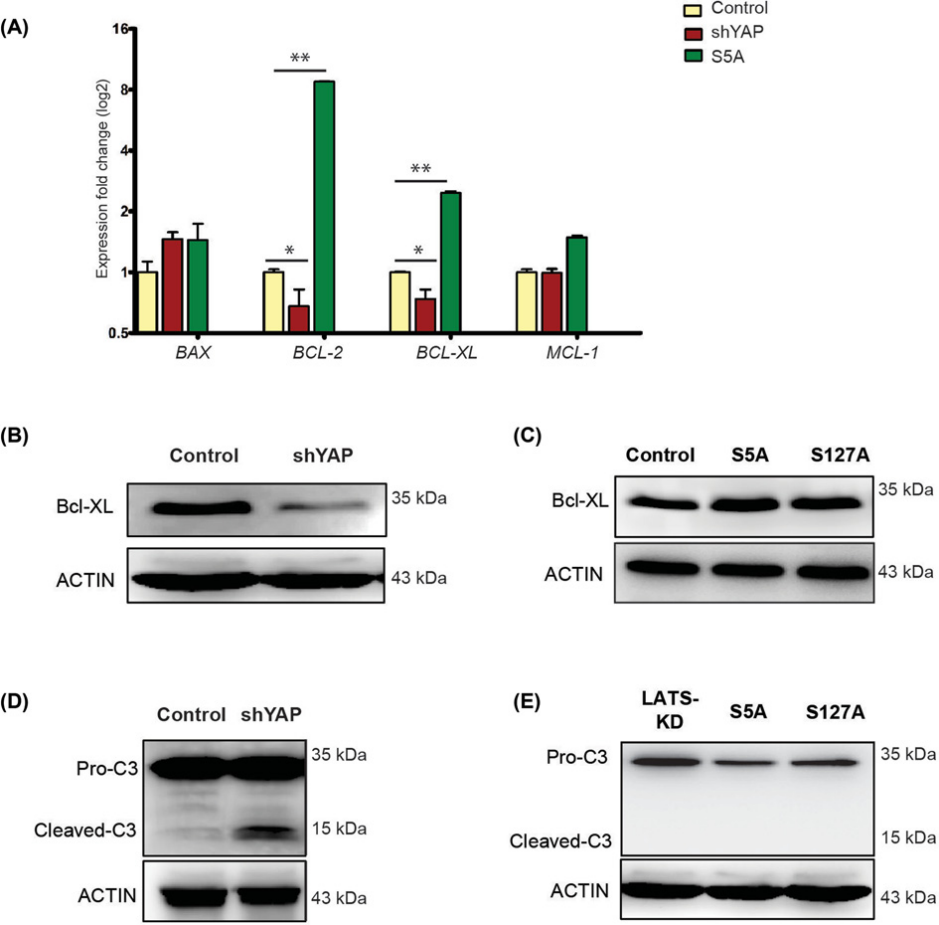
Apoptosis-associated gene expression in YAP-targeted megakaryocytes (**A**) Relative expression of pro- and anti-apoptotic-associated genes in YAP-targeted megakaryocytes by genetic or pharmaceutical molecule (*P<0.05, **P<0.01; Mann–Whitney *U* test)*.* Representative picture from three independent experiments of Western-blot analysis for *Bcl-XL* antiapoptotic gene and ACTIN in (**B**) MEG-01-shYAP, and (**C**) MEG-01-S5A and MEG-01-S127A. (**D**) Western-blot analysis for caspase-3 activity in MEG-01 cells depleted of YAP. (**E**) Western-blot analysis for caspase-3 activity in MEG-01 cells overexpressing YAP. Full Western blot images associated with Figure 3 can be found in Supplementary Figures S6–8.

Apoptosis has been described in megakaryocytes, and was found to be more prominent in mature megakaryocytes than in immature cells [13–15]. Expression of anti-apoptotic protein, such as BCL-2 and Bcl-XL, was found in immature megakaryocytes [16,17]. YAP knockdown MEG-01 cells (shYAP) and YAP overexpressing MEG-01 (YAP^S5A^) cells were subjected to anti-apoptotic gene expression analysis. Naïve MEG-01 cells served as a control. We observed a significant reduction of the anti-apoptotic genes *BCL-2* and *Bcl-XL* in YAP knockdown cells, whereas significant up-regulation of these genes was observed in YAP overexpressing cells (Figure 3A). To study the expression of apoptosis gene at the protein level, immunoblot for Bcl-XL anti-apoptotic gene was performed. Our results showed a reduction of Bcl-XL in YAP knockdown treatment (Figure 3B). However, up-regulation of Bcl-XL was observed in YAP overexpressing cell line (YAP^S5A^). The same result was observed when another mutated YAP (YAP^S127A^) was overexpressed (Figure 3C). These results suggest that expression of YAP inhibits megakaryocyte maturation.

Activation of pro-apoptotic factors, such as caspase-3, was reported in late-state differentiation of mature megakaryocytes and during platelet shedding [16,18]. Immunoblot for caspase-3 activity was performed in YAP-depleted cells. Activation of caspase-3, as determined by the appearance of their cleaved forms, was observed in YAP knockdown cells (Figure 3D). In sharp contrast, cleaved caspase-3 was not observed in YAP overexpressing MEG-01 cells (Figure 3E). Collectively, these results suggest that YAP may be a gate keeper for megakaryocytes undergoing late-stage maturation. More specifically, when YAP is reduced, cells proceed to maturation and produce more platelets, while overexpression of YAP increases CD41^+^ cells, but these cells are not producing platelets.

### PLP yields are enhanced when using a drug that targets YAP

**Figure 4:**
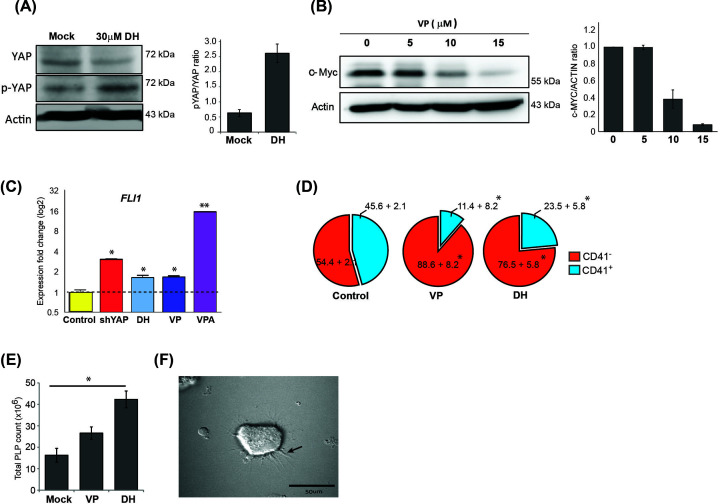
Pharmaceutical molecule targeting YAP induces platelet production (**A**) Expression of YAP and p-YAP after MEG-01 megakaryocytes were treated with 30 μM dobutamine (DH). (**B**) Expression of YAP target gene (c-*Myc*) after treatment of MEG-01 cells with various concentrations of VP. For (A and B) immunoblot signals were quantified by densitometry, and the mean of three independent experiments (one of which is shown here) was normalized to the loading control. The ratio of p-YAP to YAP was calculated as the fold difference relative to non-treated control, and the result is presented as pYAP/YAP ratio. The ratio of c-MYC was calculated as the fold difference relative to ACTIN, and the result is presented as c-MYC/ACTIN ratio. (**C**) Relative expression of a megakaryocyte-specific gene (*FLI1*) in each sample. (**D**) Pie chart showing the proportion of CD41^−^ and CD41^+^ cells in control, VP-, and DH-treated MEG01 cells (representative data from 3 independent experiments with 50,000 recorded events per experiment). (**E**) PLP population in MEG-01 treated with VP or DH was counted using an automated complete blood count (CBC) analyzer (mean ± standard deviation [SD]; *n*=3) (*P<0.05, Mann–Whitney U test). Dimethyl sulfoxide (DMSO)-treated cells served as a mock control for all experiments. (**F**) DH-treated MEG-01 cell-derived PLPs in a microplatelet aggregation assay. Activated PLPs showed irregular shapes with many protruding pseudopodia (arrow). Full Western blot images associated with Figure 4 can be found in Supplementary Figures S9 and 10.

Small molecules and drugs are more effective than direct genetic manipulation for generating large-scale *in vitro* platelet production. Hippo signaling activity can be suppressed by molecules that target different proteins in the Hippo pathway [19–23]. We used the YAP inhibitors dobutamine (DH) and verteporfin (VP) in culture to facilitate thrombopoiesis by targeting YAP. DH is known to inhibit YAP activity by phosphorylating YAP, which inhibits translocation of YAP into the nucleus, which prevents its interaction with its target genes [20]. To confirm dobutamine’s efficacy, MEG-01 cells were treated with doses ranging from 1 to 30 µM, and then the cells were collected for target gene analysis. Increase in p-YAP band intensity and reduction of YAP were observed in cells treated with 30 μM DH (Figure 4A).

VP in the absence of light activation inhibits interaction between YAP and TEAD, thus preventing expression of their downstream target genes [21]. We treated megakaryoblastic cells with 5–15 μM of VP while using reduction of the YAP/TEAD target gene c-Myc as the reporter [24,25]. Reduced expression of c-Myc was observed in a dose-dependent manner, which suggests reduction of YAP activity in treated cells. As such, 15 μM VP was selected for use in subsequent experiments (Figure 4B).

Expression of *FLI1* was shown to be critical for late megakaryopoiesis [26], and its overexpression can inhibit erythroid lineage development [27,28]. Recent study in induced pluripotent stem cells (iPSCs) found that *FLI-1* overexpression improved iPSC-derived megakaryocyte differentiation yield and its function [29]. VPA can induce maturation of megakaryocytes and induce PLP production [30]. To determine the effects of DH and VP on megakaryocyte differentiation, treated MEG-01 cells were collected and the expression of the megakaryocyte-specific gene *FLI1* was assessed. Significant up-regulation of *FLI1* transcripts was found in VPA-treated cells (Figure 4C). Up-regulation of *FLI1* was also found in each case where YAP activity had been inhibited, including in shYAP-, DH-, and VP-treated MEG-01 cells (Figure 4C). These results suggest that reduction of YAP activity induces megakaryocytic cell maturation.

To determine the effect of VP and DH on megakaryopoiesis, we compared expression of the CD41 cell-surface marker between treated and untreated cells. Interestingly, the results of flow cytometric analysis showed a significant reduction of CD41^+^ cells in the treated groups when compared with control (11.4 ± 8.2%, 23.5 ± 5.8%, and 45.6 ± 2.1%, respectively; *P*<0.05; Figure 4D). To determine the efficiency of PLP production, 2.5 × 10^6^ cells were cultured in 30 ml culture media supplemented with 10 μM VP or 30 μM DH for 3 days before PLPs were collected and counted by CBC. We found that both VP and DH treatment results in increased PLP production when compared with control (26.6 ± 2.9 × 10^6^, 42.3 ± 3.9 × 10^6^, and 16.3 ± 3.2 × 10^6^, respectively; Figure 4E). Thus, inhibition of YAP activity by VP or DH influences CD41^+^ cells to produce PLPs by 1.6- and 2.6-fold over the control value, respectively (Figure 4 and Table 1).

**Table 1:** Absolute number of platelet-like particles (PLPs) produced in verteporfin (VP) and dobutamine (DH) treatment

Condition	Total cells (×10^6^)	Total platelets (×10^6^)	Cell:platelet ratio	Fold to control
Mock	2.5	16.3 ± 3.2	1:6.5	1.0
VP	2.5	26.6 ± 2.9	1:10.6	1.6
DH	2.5	42.3 ± 3.9	1:16.9	2.6

To determine platelet function, PLPs from each group were retrieved and subjected to a platelet microaggregation assay. A representative picture in Figure 4F shows an aggregated clump of 30 μM DH treatment-derived PLPs. After thrombin activation, PLPs showed an irregular shape with many protruding pseudopodia (arrow) and form aggregates, as shown in Figure 4F. These results demonstrate that the targeting of YAP in MEG-01 cells by small molecules can enhance PLP production, and that the produced PLPs are able to perform their expected function. This finding suggests the use of small molecule targeting of YAP in MEG-01 cells for the mass *in vitro* production of platelets for transfusion.

### Expression level of YAP is essential for megakaryopoiesis and thrombopoiesis

**Figure 5:**
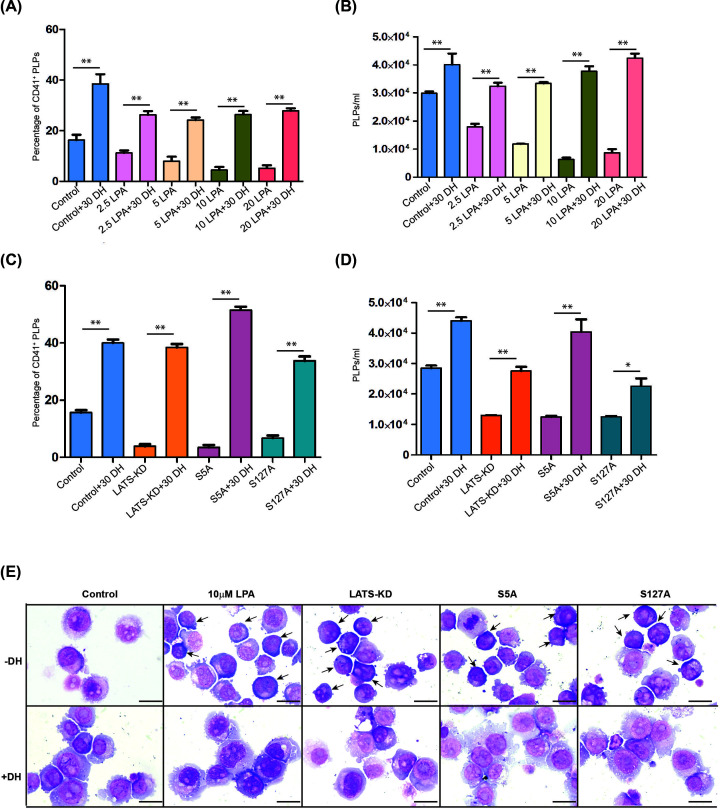
YAP tightly regulates platelet-like particle (PLP) production (**A**) Percentage, and (**B**) absolute PLP count of CD41^+^ PLPs after treatment with LPA to activate YAP, or treatment with DH to inhibit YAP. (**C**) Percentage, and (**D**) absolute PLP count after MEG-01 cells overexpressing YAP (LATS-KD, YAP^S5A^, and YAP^S127A^ cells) were treated with DH to inhibit YAP activity (*P<0.05, **P<0.01; Student’s *t*-test). (**E**) Morphologic differences of MEG-01 between treatments using Wright staining method. Arrow indicates small cell with high nuclear-cytoplasmic ratio with basophilic cytoplasm and blue staining (bar = 20 µm).

To further confirm the function of YAP during megakaryopoiesis and thrombopoiesis, sequential treatment with two small molecules was used. A small molecule targeting LATS kinase (lysophosphatidic acid [LPA]) was used to induce YAP activity [31]. A wide range of doses of LPA (0–20 μM) were used to treat MEG-01 cells for 3 days. The results showed a reduction of PLPs in a dose-dependent manner (Figure 5A,B). However, when these cells were then treated with DH, PLP production was significantly up-regulated (Figure 5A,B). Another set of experiments was performed in genetically activated YAP cell lines, including LATS1/2-KD, YAP^S5A^, and YAP^S127A^. Low PLP production yield was observed in these YAP overexpressing cell lines; however, treatment of these cells with DH significantly increased the PLP production yield (Figure 5C,D).

We also studied the morphologic differences of MEG-01 cell line after YAP was manipulated via various methods using Wright staining. While the majority of megakaryoblasts in the control group had a similar cell size with pink cytoplasm, all YAP treatments demonstrated small cells, a high nuclear–cytoplasmic ratio, basophilic cytoplasm, and blue staining (arrow). These morphologic differences suggest that YAP expression limited the maturation of megakaryocyte by most likely reversing the fate of differentiation. However, large cell size, low nuclear–cytoplasmic ratio, and distinct blebs or pseudopods were observed in DH-treated cells. These results suggest that reduction of YAP drives maturation, which results in increased PLP production (Figure 5E).

## Discussion

We previously showed that reduction of the Hippo core kinase LATS1/2 inhibits platelet production [4]. In the present study, we further demonstrated that high expression of the Hippo pathway effector protein YAP induces MEG-01 cells to become more mature as shown by the increasing number of CD41^+^ cells, but these cells did not produce platelets. Moreover, increase in YAP expression was found to inhibit caspase-3 activity in MEG-01 cells (and vice versa), which resulted in platelet release.

The role of apoptosis in megakaryopoiesis and thrombopoiesis remains controversial [32]. Previous studies reported a substantial decrease in platelet counts due to impaired apoptosis, which prevented platelet shedding from megakaryocytes [16,17,33,34]. However, studies in conditional knockout mice with blockade of intrinsic and extrinsic apoptosis pathways showed normal platelet production in both steady state and under stress condition, which indicates that proplatelet formation is a consequence of caspase-mediated apoptosis [35,36]. An alternative mechanism of platelet production was proposed using IL-1α-dependent megakaryocyte rupture in which proplatelets are not formed, but rather are directly released from the ruptured megakaryocytic cell membrane [18]. Megakaryocyte rupture is a mechanism that could produce 20-fold more platelets than the conventional proplatelet forming process, and may occur in conditions of acute platelet need, such as inflammatory-associated response. The underlying mechanism of IL-1α-dependent megakaryocyte rupture is different from that of typical FasL-induced apoptosis since there is no evidence of phosphatidylserine (PS) exposure, while caspase-3 was activated. In the present study, we found that knockdown of YAP results in a significant increase in PLP production despite the fact that most of the cells showed low or no proplatelet formation, but caspase-3 was activated. Thus, YAP may be involved in the megakaryocyte rupture mechanism.

In the present study, we demonstrated that knockdown of YAP promotes megakaryocyte maturation. However, it appears that YAP has multifunctional roles during the late stage of maturation since we found that YAP expression is required for cell differentiation, and reduction of YAP is essential for platelet release. We, therefore, propose that YAP may serve as a gatekeeper for megakaryocytes undergoing late stage of maturation and platelet release.

In summary, the dynamic manipulation of YAP via small molecules could significantly positively influence the *in vitro* production of platelets. Initial overexpression of YAP positively affects megakaryocyte proliferation and differentiation, and subsequent down-regulation of YAP leads to maturation and the release of PLPs.

## Supplementary Material

Supplementary Figures S1-S10

## Abbreviations

iPSC: induced pluripotent stem cell
LPA: lysophosphatidic acid
PLP: platelet-like particle
VP: verteporfin
VPA: valproic acid
