# Supplementary material for: Effect of YAP/TAZ on megakaryocyte differentiation and platelet production
Source: Biosci Rep. 2020 Aug 20;40(8):BSR20201780. doi: 10.1042/BSR20201780 (PMC7441484; doi:10.1042/BSR20201780)

Supplementary Figure 1 (associated with Figure 1A)

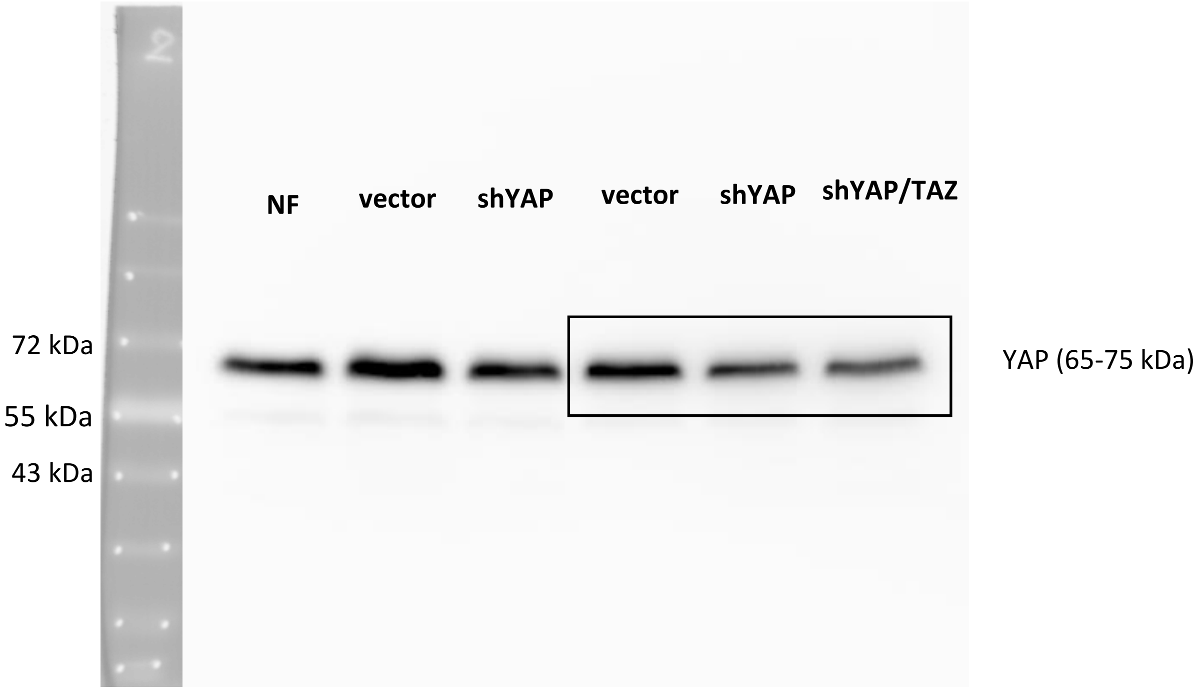

NF = non-transfected

Supplementary Figure 2 (associated with Figure 1A)

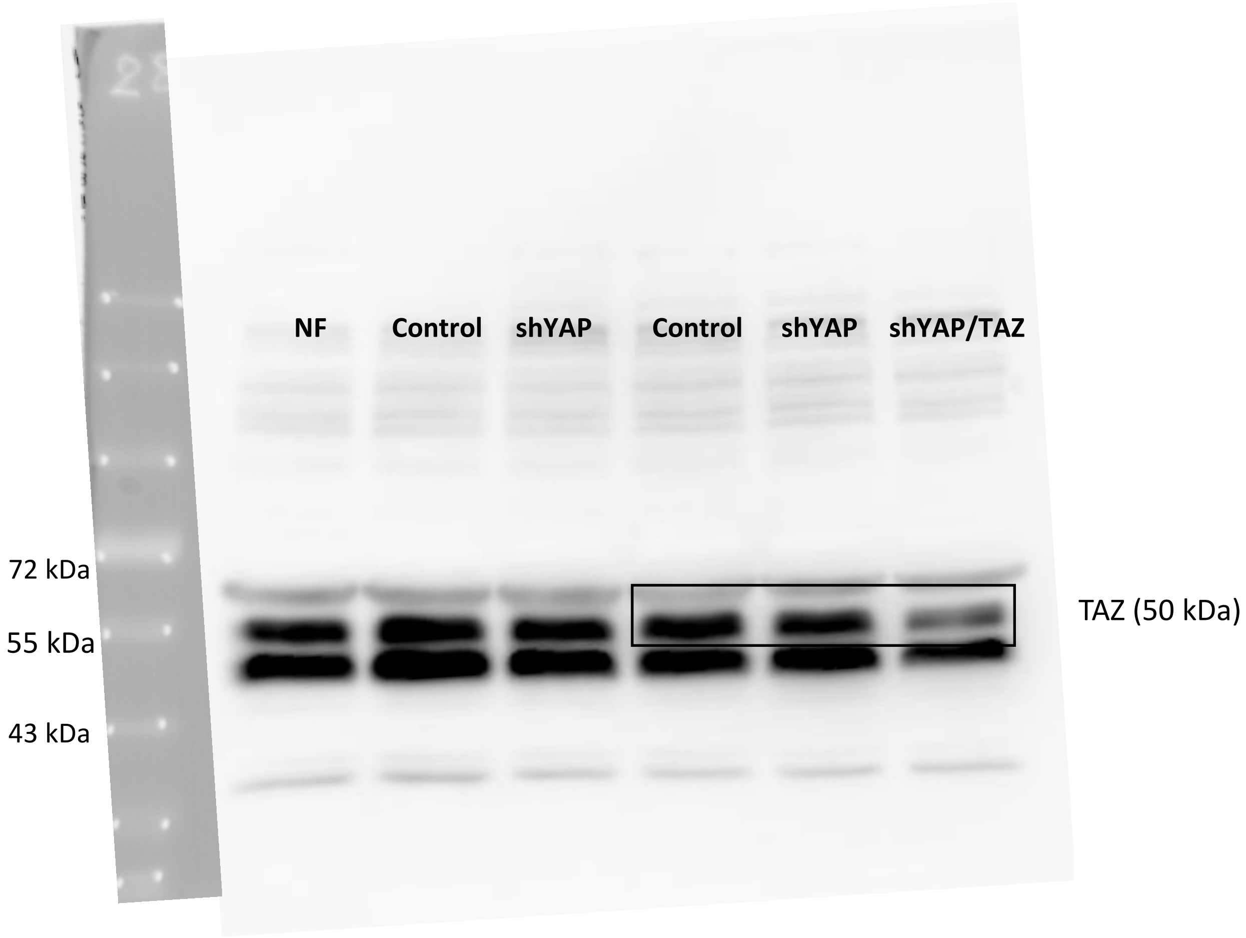

Supplementary Figure 3 (associated with Figure 1B)

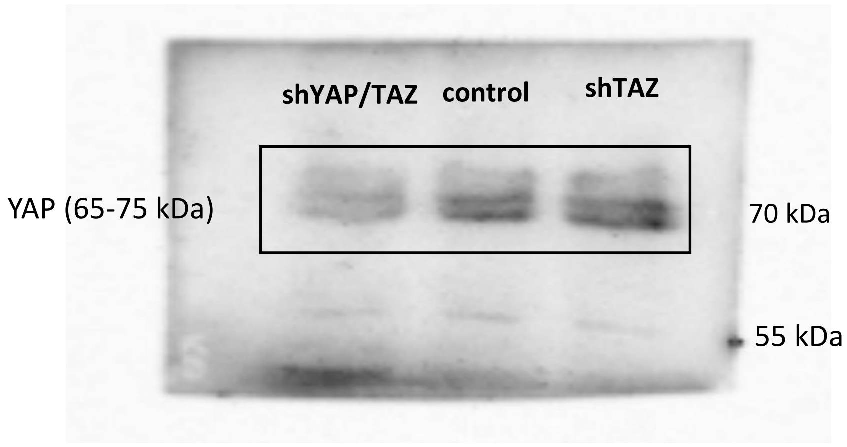

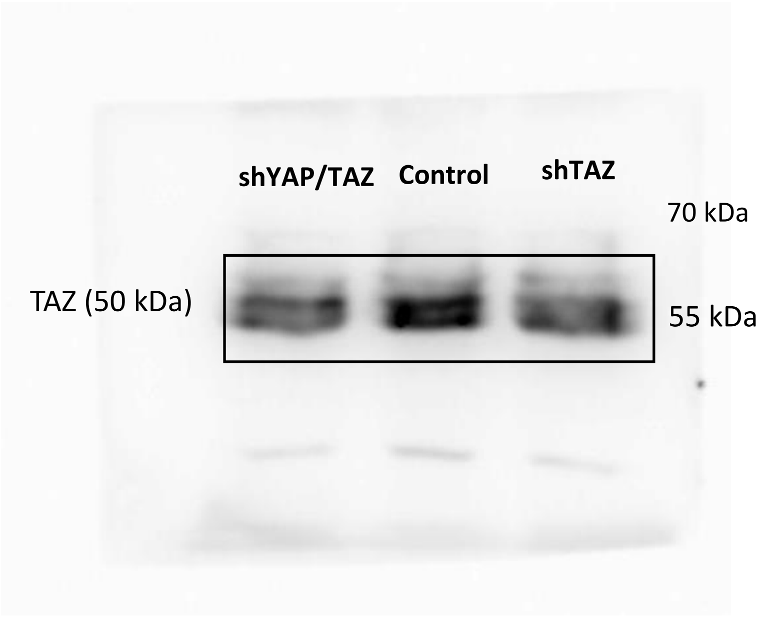

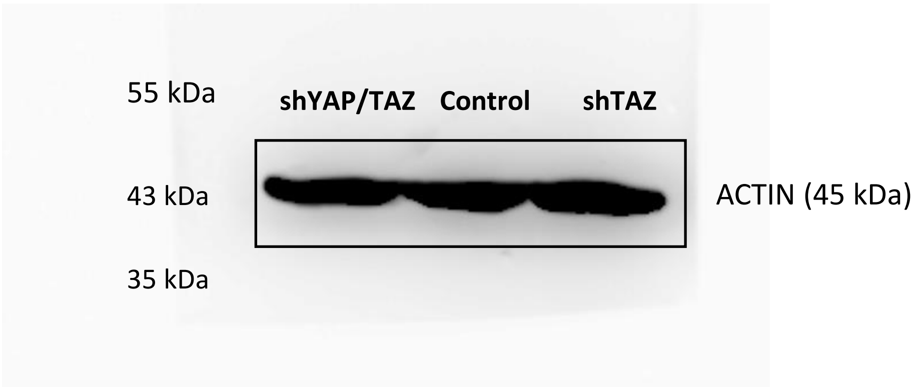

Supplementary Figure 4 (associated with Figure 2A)

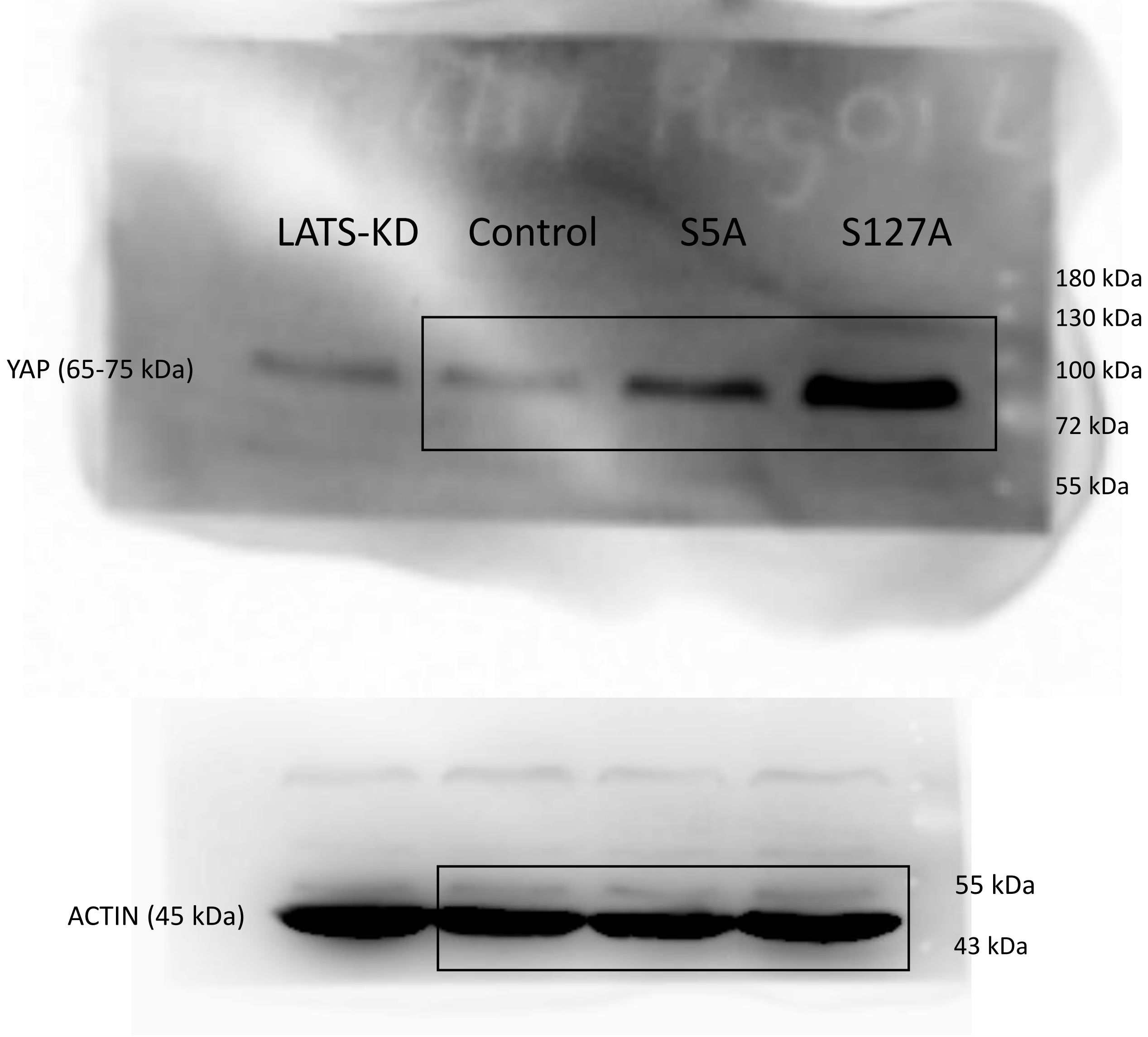

Supplementary Figure 5 (associated with Figure 2B)

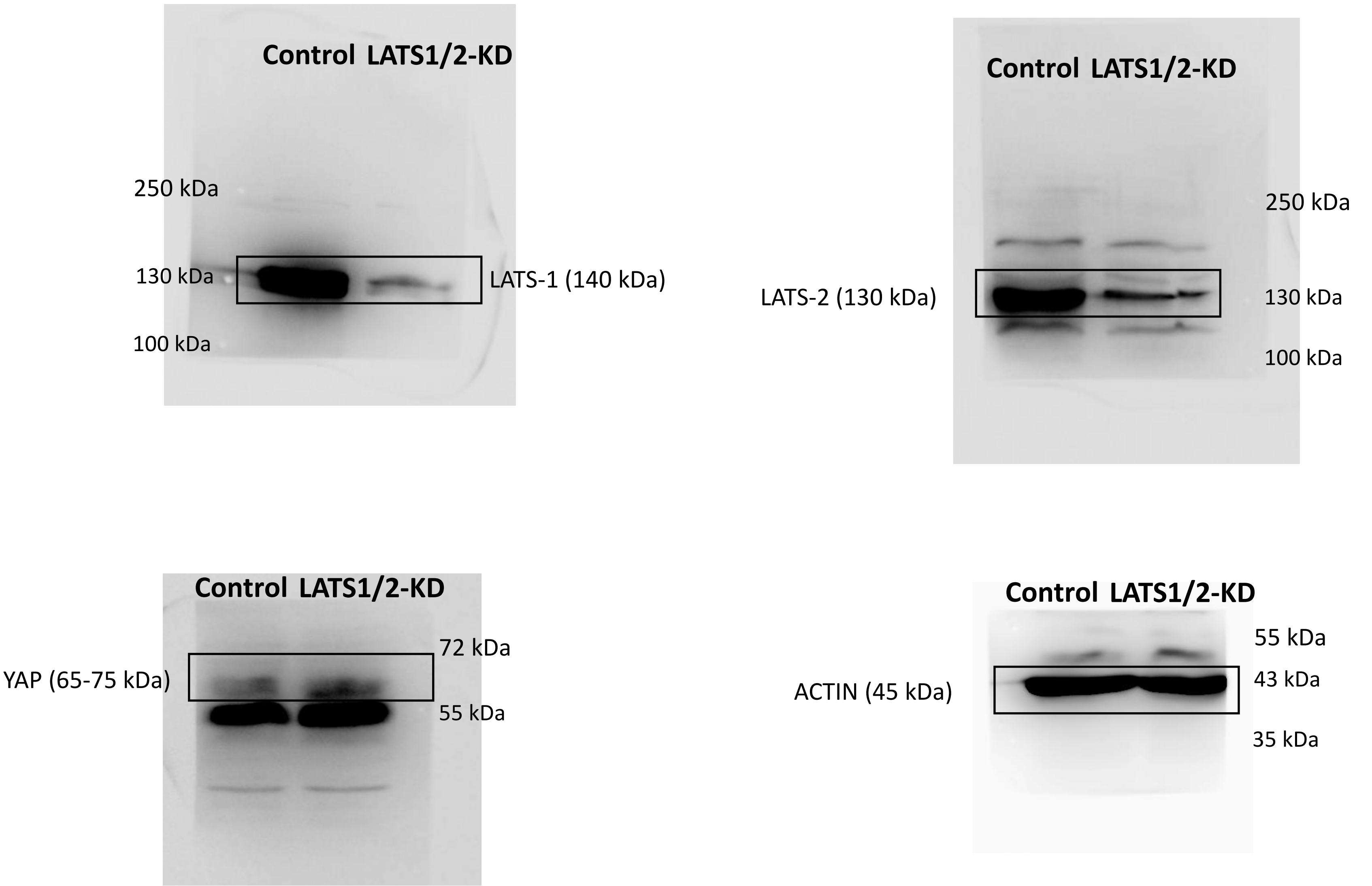

Supplementary Figure 6 (associated with Figure 3B)

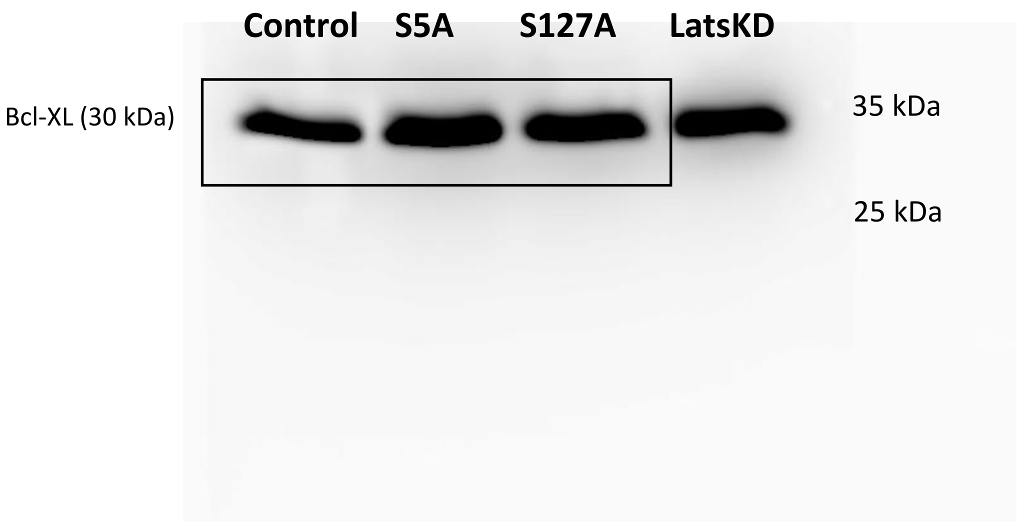

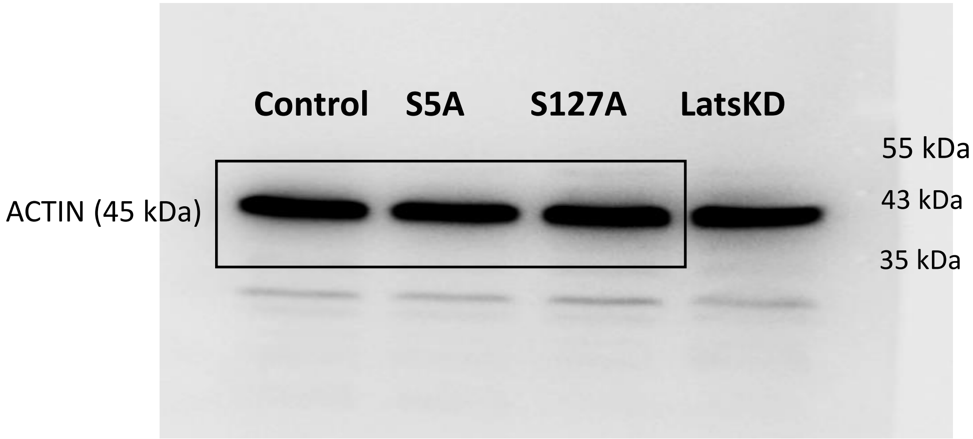

Supplementary Figure 7 (associated with Figure 3D)

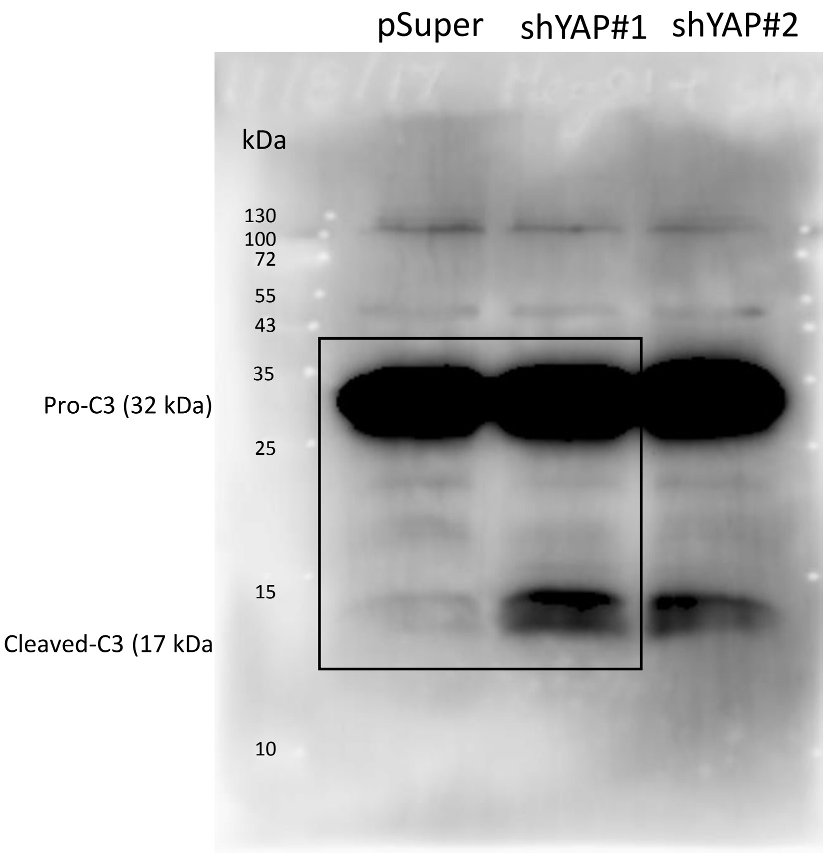

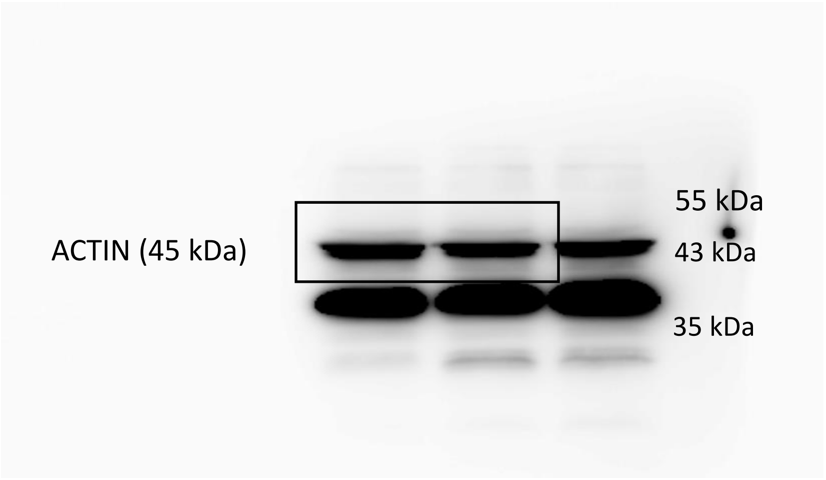

Supplementary Figure 8 (associated with Figure 3E)

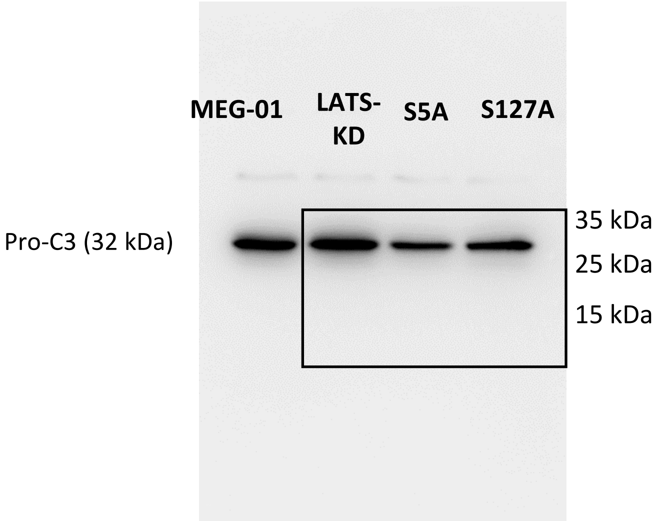

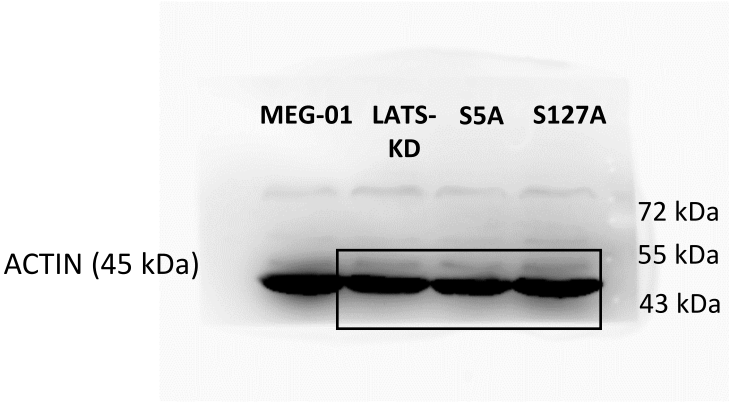

Supplementary Figure 9 (associated with Figure 4A)

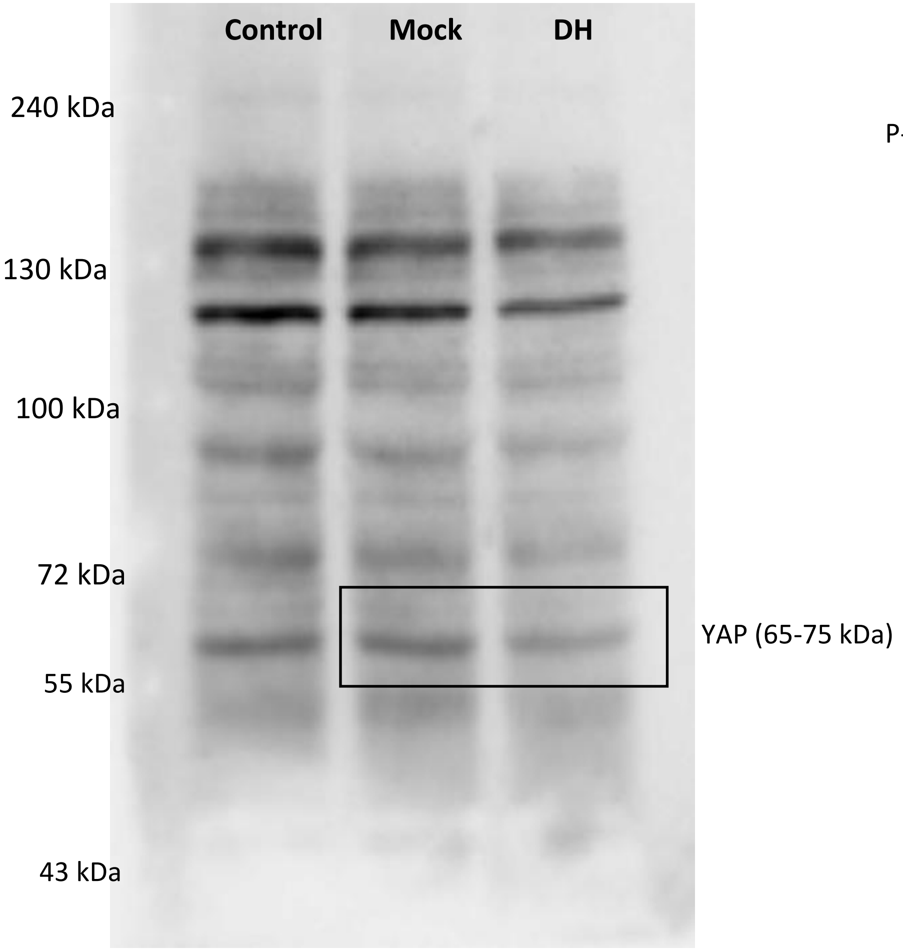

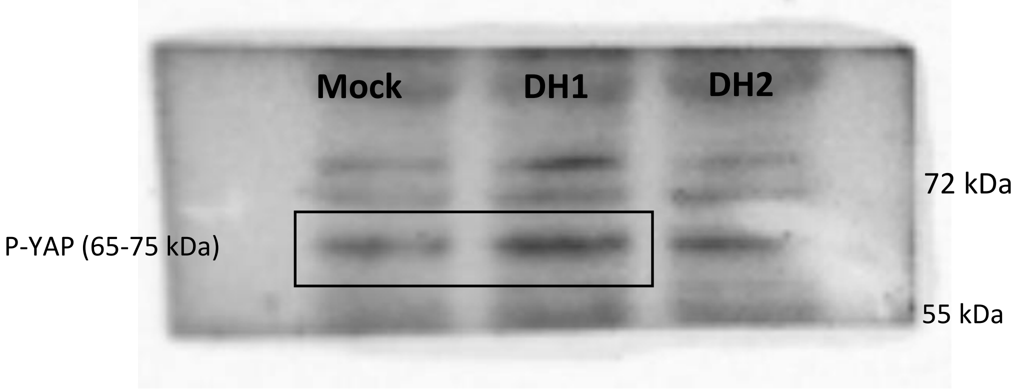

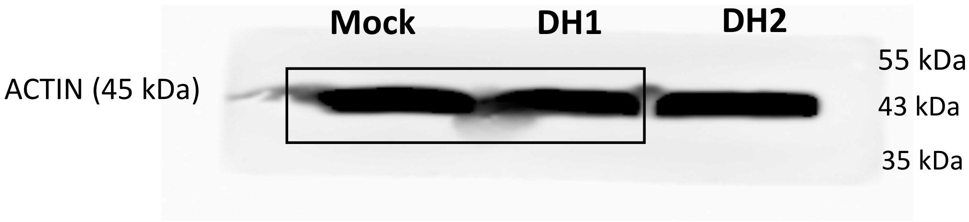

Supplementary Figure 10 (associated with Figure 4B)

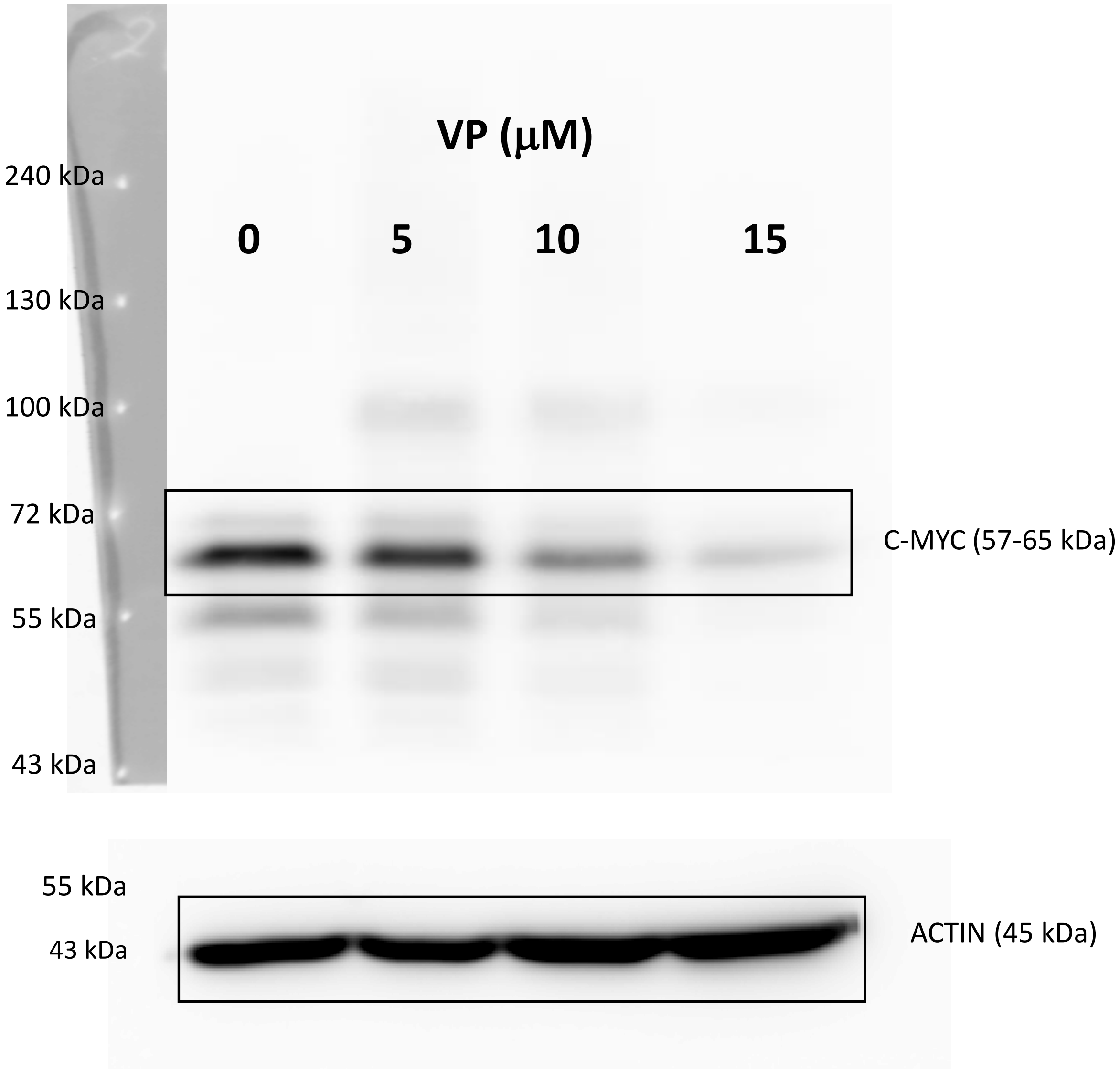

Supplement: Supplementary Figures S1-S10 [file BSR-2020-1780_supp.pdf]
